# Effect of chlorhexidine pretreatment on bacterial contamination at rhinoplasty field

**DOI:** 10.1186/s40064-016-3679-y

**Published:** 2016-12-30

**Authors:** Shin Hye Kim, Keng Lu Tan, Sang Yeon Lee, Dae Woo Kim, Sue Shin, Hong-Ryul Jin

**Affiliations:** 1 Department of Otorhinolaryngology-Head and Neck Surgery, Korea University Medical Center, Korea University College of Medicine, Seoul, Korea; 2 Department of Otorhinolaryngology-Head and Neck Surgery, University Malaya, Kuala Lumpur, Malaysia; 3 Department of Otorhinolaryngology-Head and Neck Surgery, Boramae Medical Center, Seoul National University College of Medicine, 20 Boramae-ro 5-gil, Dongjak-gu, Seoul, Korea; 4 Department of Laboratory Medicine, Boramae Medical Center, Seoul National University College of Medicine, Seoul, Korea

**Keywords:** Rhinoplasty, Pretreatment, Chlorhexidine, Culture, Infection

## Abstract

**Background:**

This study investigated on bacterial contamination of the rhinoplasty field. The effect of preoperative chlorhexidine treatment on decreasing bacterial contamination in the rhinoplasty field is examined.

**Methods:**

Thirty patients who underwent rhinoplasty were block randomized into a chlorhexidine, regular-soap, or control group comprising ten participants each. The chlorhexidine group was subjected to chlorhexidine showering, shampooing, and facial-cleansing 12 h prior to the operation. The regular-soap group was subjected to cleansing with regular soap, and the control group did not have any skin pretreatment. Bacterial cultures were done 12 h preoperatively from nasal cavity and perinasal skin, immediately preoperatively from perinasal skin and at 1 and 2 h intraoperatively from operation field. Culture results were compared between the three groups, according to operation time, or whether infection-prone procedure was performed.

**Results:**

The bacterial species and colony-forming unit numbers at preoperative nasal cavity and perinasal skin were similar. In all three groups, *Coagulase*-*negative staphylococcus* was the most common bacteria found in the rhinoplasty field. The numbers of *Staphylococcus aureus* and *Corynebacterium* decreased rapidly after preoperative chlorhexidine treatment. The infection-prone procedure was associated with increased bacterial numbers over time during the operation. In all three groups, there was no postoperative infection in a follow-up period of 6 months.

**Conclusion:**

Rhinoplasty is confirmed as a clean contaminated operation with skin flora consistently found in the operation field. Chlorhexidine pretreatment in rhinoplasty patients has a tendency to decrease the numbers of *Staphylococcus aureus* and *Corynebacterium* on the perinasal skin.

**Level of evidence:**

Randomized controlled trial, Level I.

## Background

Asepsis was first introduced in 1860 into the practice of surgery. It revolutionized the practice of surgery from frequent infection and death to prolonging life and improving quality of life (Digison 2007). Since then, it has become an intensive pursuit to eliminate surgical site infection (SSI). SSI is associated with various undesired complications that may hinder the surgeon’s best effort to obtain a good result. Septoplasty or rhinoplasty is considered a clean contaminated operation (Durmaz et al. 2011). SSI in rhinoplasty could result in failure of implant and severe scarring of the nose, resulting in possible cosmetic and functional disaster.

Currently in the practice of rhinoplasty, the risk of SSI varies depending on the status of previous rhinoplasty, implant materials, and surgical techniques and is reported to be under 1% (Warnke et al. 2010; Won and Jin 2010; Abifadel et al. 1990). The prevention of infection is even more important in Asian rhinoplasty as the frequent use of alloplastic implant renders it more vulnerable to infection (Won and Jin 2010; Loyo and Ishii 2013). Although antibiotics administered pre- and post-operatively can reduce SSI rate in rhinoplasty patients (Warnke et al. 2010), there is still a substantial risk of infection. Due to the disastrous consequences of SSI in rhinoplasty, it is pertinent to investigate on additional ways to reduce bacterial load intraoperatively.

One of the ways to achieve this is to administer preoperative skin sterilization. Skin pretreatment, among others, is important as the main source of infection is likely to be direct inoculation of the patients’ own microflora, especially from the skin and the manipulated site (Holt et al. 1987; Rodrigues and Simões 2013). Antiseptic agents such as povidone-iodine, chlorhexidine gluconate and alcohol are most commonly used for skin pretreatment (Yammine and Harvey 2013). Studies on the effect of skin pretreatment on prevention of postoperative infection have been done mainly in orthopedic, gastrointestinal or gynecologic surgery but not in rhionoplasty (Yammine and Harvey 2013; Darouiche et al. 2010; Swenson et al. 2009).

So far there has been no study performed to characterize the bacterial population in the rhinoplasty surgical field and bacterial contamination before and after skin pretreatment. The objectives of this study are to characterize the bacterial population preoperatively and intraoperatively during rhinoplasty and to examine the effect of chlorhexidine in decreasing postoperative infection.

## Methods

The patients receiving rhinoplasty in the department of otolaryngology at Boramae Medical Center from June 2013 to December 2013 were evaluated. Thirty hospitalized patients who provided informed consent were included in this study, ten each in the chlorhexidine group, the regular-soap group and the control group. Patients who had active infection (e.g. acute rhinosinusitis), chlorhexidine allergy, antibiotics treatment in the last 30 days prior to surgery and previous rhinoplasty were excluded from the study. This was approved by the Institutional Review Board of Boramae Medical Center (IRB No: 26-2013-6).

To prevent selection bias, block randomization was performed to determine the sequence of chlorhexidine, regular-soap, and control group. The patients were assigned to the groups based on the order of hospitalization. The chlorhexidine group was subjected to shampooing, showering, and facial-cleansing with chlorhexidine gluconate solution (Hexidine^®^, Microshield 4, Johnson & Johnson Medical, North Ryde, Australia, 4% chlorhexidine gluconate with detergent, emollient, and moisturizer) 12 h prior to the surgery. The regular-soap group was subjected to pretreatment with regular soap provided by the hospital (Hair and body soap, LG Household & Care, Korea). The control group did not receive any pretreatment preoperatively.

**Fig. 1:**
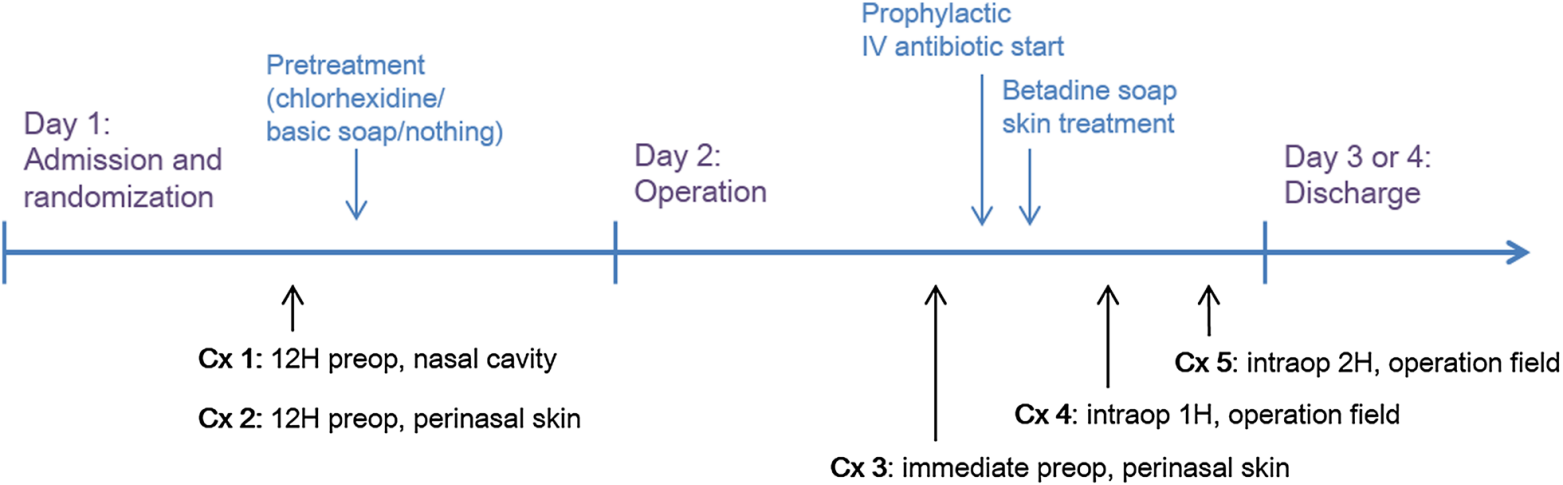
Schematic flow chart showing bacterial culture timing: five bacterial swabs (Cx1–5) are taken from one subject at four different time points

Five bacterial swabs were taken from each subject at four different point of time (Fig. 1). Twelve hours prior to the surgery, bacterial swab was taken using a sterile cotton swab from the nasal cavity and perinasal skin in all three groups before skin treatment [culture at nasal cavity (Cx1), culture at perinasal skin (Cx2)]. In the following day, swab was repeated at the perinasal area immediately before povidone-iodine (Betadine^®^) skin treatment and draping in the operation room (Cx3). At 1 and 2 h intraoperatively, bacterial swab was taken again from the dorsal cavity (Cx4 and Cx5).

During the surgery, operation field was irrigated several times with normal saline. Implant material soaked with antibiotics was used. Immediately before surgery, the patients were given intravenous cefotetan 1 g (Yamatetan^®^) and then twice a day until they were discharged from the hospital. Patients were prescribed 7 days of oral cefpodoxime (Banan^®^) 200 mg every 12 h upon discharge.

The samples were stored in a conical tube containing 1 ml of 0.9% normal saline. The samples were cultured within 6 h of collection. Two hundred microliter aliquots of each sample were inoculated onto blood agar, MacConkey agar and Sabouraud dextrose agar plates, and then these plates were incubated aerobically for 48 h at 37 °C. After 48 h of incubation, the numbers of colony-forming unit (CFU) on each plate were recorded, and the bacterial species were identified (VITEK II). When the identified bacteria exceeded 300 CFU, it was simply recorded as 300 CFU.

Bacterial identification was performed according to microbial examinations standards for categorization; a small bacterial population of common nasal cavity was categorized as normal flora (Yammine and Harvey 2013). Any large bacterial populations including typically normal residents were regarded as pathogenic flora, therefore the number of those were counted and reported. Bacterial species and numbers of CFU were analyzed and compared between the groups according to the time sequence.

The number of operative procedures (septoplasty, osteotomy, septal extension graft and spreader graft) and graft material (rib cartilage, temporalis fascia or allofascia) was taken into account. The use of rib cartilage and/or temporalis fascia was defined as infection-prone procedure. The relationship between the infection-prone procedure and bacterial numbers of CFU was analyzed. Finally, patients were seen 6 months postoperatively to look out for postoperative infection. Statistical analyses were performed using SPSS 18.0 for Windows (SPSS Inc., Chicago, IL). Non-parametric Mann–Whitney U-test compared the continuous variable among the experimental, regular-soap, and control groups. The Mann–Whitney U-test also used to compare continuous variable between infection-prone procedure group and other group. Paired *t* test compared the CFU values before pretreatment with after treatment in each groups. The association of pathogen identification was estimated by calculating the relative risk and 95% confidence interval; the *p* values less than 0.05 were considered significant.

## Results

**Fig. 2:**
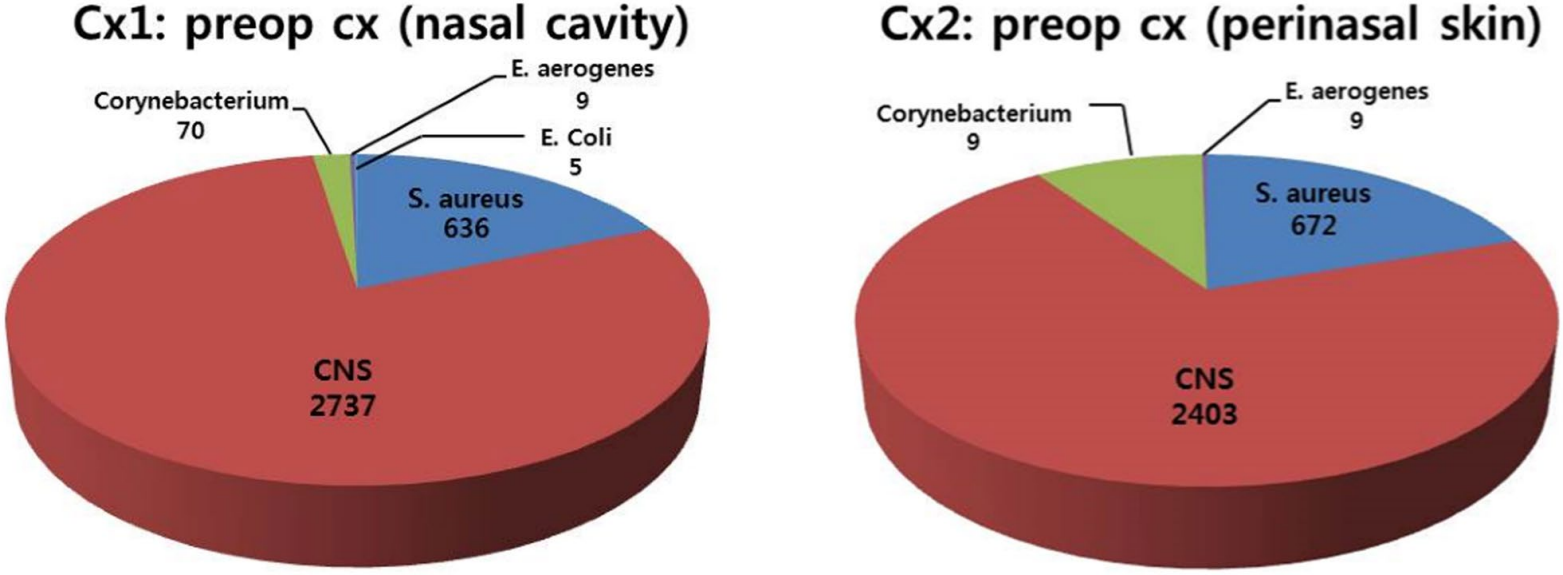
Culture results showing the numbers of CFU before skin pretreatment in total number of 30 patients: preoperative culture results at nasal cavity (Cx1) and at perinasal skin (Cx2) shows mainly *CNS*, followed by *S. aureus*, and *Corynebacterium*

The number, mean age, sex ratio and operation time of the three groups were shown in Table 1. There was no significant difference in operation time among the three groups. Preoperative bacterial distribution in total numbers of 30 patients was shown in Fig. 2. Cx1 (12H preop, nasal cavity) and Cx2 (12H preop, perinasal skin) showed similar species and numbers of bacteria.

**Table 1:** Demographics of the 30 patients comprising of three groups

Groups	Chlorhexidine (N = 10)	Regular-soap (N = 10)	Control (N = 10)	*p* values
Mean age (years)	25.4	30.2	35	0.112
Sex ratio (male:female)	9:1	4:6	6:4	0.067
Operation time (min)	168.9	172.3	151.1	0.675
Diabetes (number)	1	0	1	0.612
Smoking (number)	2	1	2	0.804

**Fig. 3:**
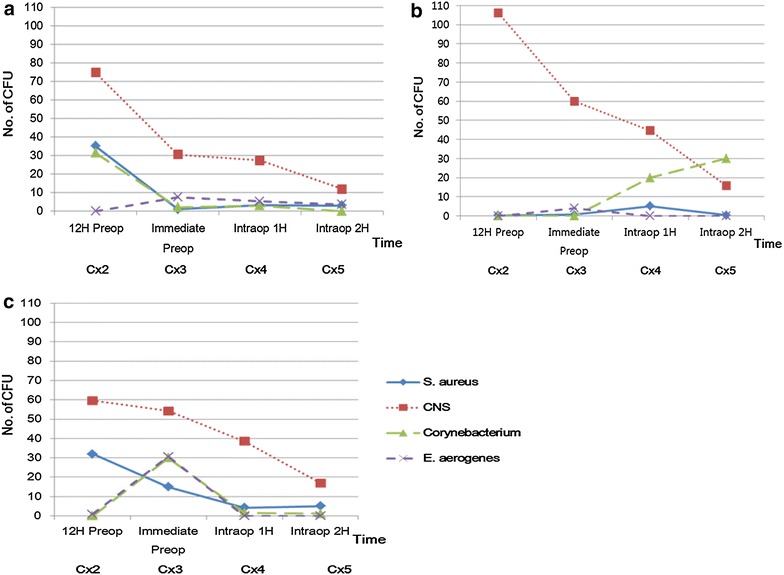
Culture results showing the numbers of CFU in the three groups: The changes of the numbers of CFU over time are different with Chlorhexidine group (**a**), regular-soap group (**b**), and control group (**c**). All the numbers of CFU are mean of ten patients in each group

Quantification of the CFU for the bacteria cultured is shown in Fig. 3. *Coagulase negative staphylococcus* (*CNS*) was the most prevalent bacteria in all three groups. Among all the pathogens identified, gram-positive pathogens including *CNS, Staphylococcus aureus (S. aureus),* and *Corynebacterium* were the main bacteria cultured around the rhinoplasty surgical site. Gram-negative pathogens including *Enterobacter aerogenes* (*E. aerogenes*), and *Escherichia coli* (*E. coli*) were rarely observed in all three groups.

Antiseptic effects of each pretreatments can be extrapolated from the comparison of Cx2 (12H preop, perinasal skin) and Cx3 (immediate preop, perinasal skin). In the chlorhexidine group, the numbers of *S. aureus* and *Corynebacterium* at Cx3 decreased to almost zero count compared to Cx2 but it was not statistically significant (Fig. 3a). In the regular-soap group, CFU number of *CNS* showed a decline but it was not statistically significant. The other bacteria were rarely contaminated state at 12 h prior to preoperatively (Cx2), so their change could not be seen at Cx3 (Fig. 3b). In the control group, the number of *CNS* and *S. aureus* slightly decreased but the numbers of *Corynebacterium* and *E. aerogenes* increased in Cx3 compared to Cx2 (Fig. 3c). On serial changes from Cx3 (immediate preop, perinasal skin) to Cx4 (intraop 1H, operation field) and Cx5 (intraop 2H, operation field), all bacterial species at all 3 groups decreased from Cx3 to Cx5 except for *Corynebacterium* of regular-soap group (Fig. 3).

**Fig. 4:**
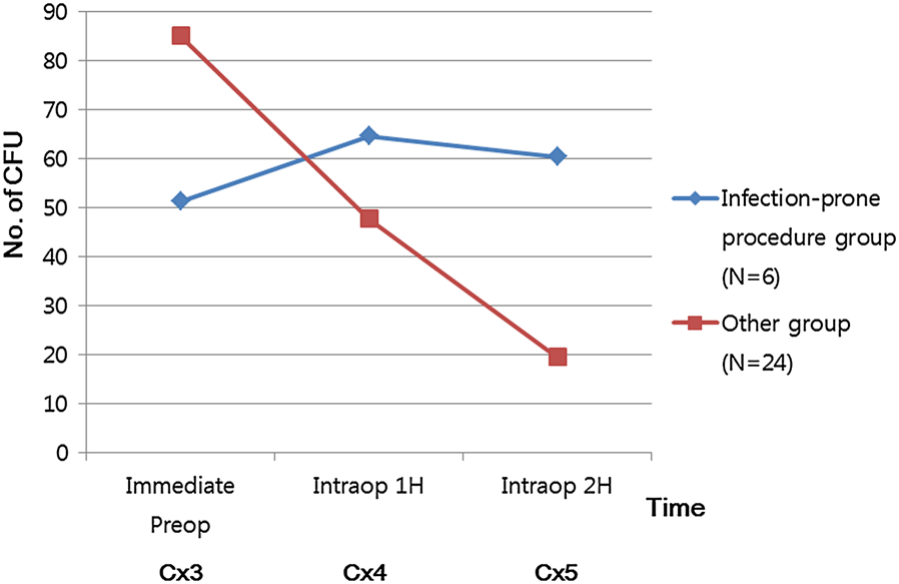
Culture results in infection-prone procedure group and other group: All the numbers of CFU are mean of patients in each group. Infection-prone procedure is defined as use of rib cartilage graft or temporalis muscle fascia

The number of operative procedures (septoplasty, osteotomy, septal extension graft, or spreader graft) and graft material used (rib cartilage, temporalis fascia, or allofascia) were similar among the three groups (Table 2). The relationship between the infection-prone operative procedure and bacterial numbers of CFU was analyzed. Infection-prone operative procedure such as use of rib cartilage or temporalis fascia was associated with increased bacterial numbers of CFU over time during the operation, but it was not statistically significant (Fig. 4). There was no postoperative infection in all 30 patients during a follow up period of 6 months.

**Table 2:** Operative procedures and graft materials used in the chlorhexidine, regular-soap and control groups

Groups	Septoplasty	Osteotomy	SEG	Spreader graft	RCG	TF	Allofascia
Chlorhexidine	10	7	5	5	3	1	0
Regular-soap	10	5	3	4	3	1	2
Control	10	9	2	7	0	0	3

The numbers are not mutually exclusive

*SEG* septal extension graft, *RCG* rib cartilage graft, *TF* temporalis muscle fascia

## Conclusion

Although this study is a prospective, randomized, and controlled study, the size of each group was not large enough to deduce statistically significant results. Future study with a larger numbers of patients will be necessary to elucidate the effect of chlorhexidine pretreatment in the prevention of SSI in rhinoplasty. In conclusion, authors found that rhinoplasty surgical field is not sterile and continuously exposed to bacterial floras of perinasal skin and nasal cavity. Chlorhexidine pretreatment shows some effect in decreasing the number of *S. aureus* and *Corynebacterium* on the perinasal skin but its effect on the prevention of postoperative infection needs further study.

## Discussion

There have been some reports about the SSI during septoplasty or rhinoplasty (Slavin et al. 1983; Silk et al. 1991; Okur et al. 2006), but there has been no study about the bacterial colonization at the rhinoplasty surgical field itself. The fact that *CNS, S. aureus, Corynebacterium*, *E. aerogenes* and *E. coli* were found in considerable amount in this study shows that rhinoplasty surgical field is not completely sterile and continuously exposed to bacteria from the perinasal skin or nasal cavity.

Povidone-iodine is commonly used as an iodophor antiseptic by destroying microbial proteins and DNA (Hemani and Lepor 2009). It can be safely used on mucous membrane surfaces, but its activity is limited by the amount of time the agent is in contact with the skin. Chlorhexidine has been used recently as a skin antiseptic. Chlorhexidine works by disrupting bacterial cell membranes and it has more sustained antimicrobial activity than other local antimicrobials because its resistance to neutralization by blood products than the iodophors (Veiga 2009; World Health Organization, Department of Reproductive Health and Research 2010). There is one report showing the effect of chlorhexidine pretreatment compared with regular-soap and no shower in reducing the bacterial contamination in breast reconstruction or liposuction (Webster and Osborne 2006). However, unlike our study, this study only identified and compared bacteria CFU after respective pretreatments without including pre-sterilization and intraoperative bacterial CFU.

Chlorhexidine has been used as a skin pretreatment agent, and it has gained popularity as a hand-scrubbing and showering antiseptic prior to surgery (Hibbard 2005). There have been studies supporting that chlorhexidine is more effective than povidone-iodine in decreasing SSI of abdominal, thoracic, or gynecologic surgery or bacteremia in neonates weighing greater than 1500 g at birth (Darouiche et al. 2010; Nuntnarumit and Sangsuksawang 2013). A meta-analysis study with 9 prospective, randomized controlled clinical trials suggested that the use of chlorhexidine for skin antisepsis, instead of povidone-iodine, would result in significant reduction in hospital-acquired infections and hospital costs (Miller et al. 2008). Combined sequential use of chlorhexidine and povidone-iodine is known to be superior to either regimen alone in skin disinfection (Langgartner et al. 2004; Guzel et al. 2009).

In this study, the perinasal skin was selected as a representative culture site proving the effect of chlorhexidine pretreatment because perinasal skin is a routinely exposed site that might contaminate the operation field during rhinoplasty. It is also an area where chlorhexidine pretreatment can easily implement its effect. The fact that the culture results from the nasal cavity, which can be another source of rhinoplasty field infection, showed similar result with the perinasal skin also supports our choice.

The decreased CFU numbers of *S. aureus* and *Corynebacterium* at the perinasal area in chlorhexidine group compared to other groups shows that the chlorhexidine pretreatment is effective in reducing the *S. aureus* and *Corynebacterium* contamination although a small sample size did not show a statistical significance. It is meaningful because *S. aureus* is a commonly found and virulent organism in postoperative wound infection. *S. aureus* is found in about 50% of all healthy persons in the nasal vestibule (Rettinger 2007), and the incidence of *methicillin resistant S. aureus* (*MRSA*) colonization is reported as approximately 0.8% in the US population and 0.7% in Australia community, and 3% in Pakistani community (Anwar et al. 2004). A number of different organisms, most prominently *S. aureus*, *Streptococci*, *Anaerobes*, and *Corynebacteria* are identified in oral and nasal mucosa. Besides oral and nasal cavity, since exposed areas such as the face, neck, and hands have higher total numbers of bacteria including more transient bacteria such as *group A streptococci*, skin pretreatment of these exposed area is also important to prevent contamination in surgeries of these areas.

From Cx3 to Cx5, all bacteria including *CNS* showed a steady decline in CFU except for *Corynebacterium* in regular-soap group. Possible explanations are the effect of preoperative intravenous antibiotics, betadine skin treatment and the irrigation of the operation field during the surgery. Preoperative and postoperative antibiotics and povidone-iodine skin preparation are routinely implemented procedures in our practice of rhinoplasty. Even though our study showed decrease in the perinasal skin colonization of *S. aureus* after chlorhexidine pretreatment, its preventive effect on postoperative infection is difficult to determine because there were no clinical infection case in all three groups.

In this study, there was a hypothesis that infection-prone procedure such as use of rib cartilage or temporalis fascia in rhinoplasty may increase the bacterial numbers during the operation. The bacterial colonies of nasal cavity or chest may move to rhinoplasty surgical field, that is, intradorsum. No patients in the control group used rib cartilage or temporalis muscle fascia while they were used in 3 patients of hexidine group and 3 patient of regular-soap group. Although the bacterial numbers of CFU increased in groups with infection-prone procedure, it was not statistically significant. This may be attributed to small numbers of patients. Other possible explanations are the masking effect of intravenous antibiotics, effect of shampooing or shower with chlorhexidine in chlorhexidine group, or the irrigation of the operation field during the surgery.
